# Changes in cancer incidence and mortality in England and Wales and a comparison of cancer deaths in the major developed countries by age and sex 1979–2002 in context of GDP expenditure on health

**DOI:** 10.3332/eCMS.2008.80

**Published:** 2008-07-28

**Authors:** C Pritchard, T Hickish

**Affiliations:** 1 Professor in Psychiatric Social Work, School of Health & Social Care, Bournemouth University, UK, and Emeritus Professor, School of Medicine, University of Southampton, UK; 2 Consultant Medical Oncologist, Dorset Cancer Centre, UK, and Visiting Professor, School of Health & Social Care, Bournemouth University, UK

**Keywords:** comparison, cancers, deaths, incidence, age, sex

## Abstract

**Background:**

The successful treatment of cancer is a major health and political issue for England and Wales and the major developed countries (MDCs). All malignancy deaths by age and sex are analysed to determine how successful the MDCs were in reducing cancer mortality between the end points of 1979–81 and 2000–2, and whether there was any association between each nations ‘gross domestic product expenditure on health’ (GDPEH) and the reduction in their cancer deaths.

**Method:**

Incidence of cancer in England and Wales was examined for 1979–80 to 2003–4 to highlight the extent of the problem. The cancer mortality rates for England and Wales were compared with each MDC by age and sex, using ‘WHO all malignancies mortality rates’ for the periods of 1979–81 and 2000–2, and tests of significance were made. The GDPEH for each MDC was examined for 1980–2002, and Spearman rank-order correlations calculated to explore any association between declining cancer deaths and the GDPEH of each MDC.

**Results:**

1. Men’s All Age malignancy incidence in England and Wales rose 48% and women’s 51%, with notable rises for females aged 15–34 and 55–74 years.
2. Every MDC increased its GDPEH substantially; it rose to 9.3% in the United Kingdom, but the United Kingdom still remains eighth of the ten MDCs and below the MDC average (9.85%).
3. The average number of cancer related deaths for men in England and Wales (15–74 years) was third highest in 1979–81, but fell to eighth by 2000–2. This decline was significantly greater than in seven other MDCs. Average female death rates in England and Wales were highest both in 1979–81 and in 2000–2, but declined significantly more than most MDCs in every age band from 35 to 74 years.
4. There was a significant correlation between reduced deaths and the level of GDPEH of each nation.
5. Male death rates declined significantly more than that of female in each MDC, with the exception of Japan and Spain.

**Conclusions:**

The rising incidence in cancer-related deaths poses a problem for every MDC, and the poorer women’s results should be a matter of concern for most MDCs. The reduction in cancer deaths reflects well on frontline services, and the significant association between reduced cancer mortality and increased GDPEH is encouraging, but still a challenge for governments, especially if the incidence continues to rise.

## Background

Cancer incidence in developed countries has been rising for decades [1–3] and governments have responded by making major commitments to reduce cancer morbidity and mortality [4,5], raising the question: ‘How effective have the different countries been?’

Previous studies of ‘effectiveness’ concentrated on five-year cancer survival rates from the 1990s where, in comparison, England and Wales did poorly [6–9], although these studies were too early to examine any effect of the new NHS investment into cancer services [4,10].

The problem with survival studies is that there can be variations in the time of diagnosis [3,6], and furthermore, in some studies, the rates are not based on absolute survival rates but rather compared with the survival of the general population [9].

This study takes an alternative approach by analysing adult cancer death rates, which shows some gains and some losses [11–13], using the latest standardized WHO data between the end points of 1979–81 and 2000–2 [14], to explore the effectiveness of England and Wales in reducing cancer mortality compared to the other major developed countries (MDCs) by age and sex.

This is set within the context of gross domestic product expenditure on health (GDPEH) of each MDC, as it is recognized that efforts to reduce cancer mortality have substantial national costs reflected by the GDPEH [4,5].

This hypothesis-generating study has three general null hypotheses that between the endpoints of 1979–81 and 2000–2, there will be no statistically significant differences:

1. in ‘all malignancy cancer’ death rates in England and Wales and the other nine MDCs by age and sex;
2. between the gender in each MDC;
3. no association between reduced cancer mortality and increases in national GDPEH.

These hypotheses are explored within two contextual frameworks, the changing incidence of cancer in England and Wales [3,15] and the differential national commitments seen in the GDPEH by each of the MDCs between 1980 and 2003 [16].

## Method and design

The problem: the incidence of cancer for the two-year period 1979–80 is compared with the latest data available for the two-year period 2003–4 from Anglo-Welsh official statistics [3,15], which indicates the continuing extent of the problem.

All cancer registrations are reported by age and sex but because of the possible influence of increased screening on female rates [17], the focus was on malignancies (coded C00-97) to match the mortality categories in ICD 9th and 10th editions [14,18].

National responses: the international response to the growing incidence of cancer is reflected in national GDPEH data [16]; the changes between 1980 and 2003 were analysed, and an annual average and percentage increases calculated.

The possible influence of increased GDPEH on cancer death rates was determined by calculating the death rates proportionate to the level of GDPEH and the percentage of GDPEH increase. Any association between increased GDPEH and declines in cancer deaths was tested by a Spearman rank-order (Rho) correlation for the combined malignancy death rates of both sexes and each sex and the percentage change in deaths and GDPEH. It is recognized that any significant positive correlation is not necessarily causal but can be considered as indicative of a link between expenditure on health and reduction in cancer deaths.

Outcomes: the outcome of national efforts to effectively prevent and treat cancer can be seen in WHO mortality statistics drawn from the latest available standardized data based upon ‘all malignant neoplasm’ deaths (coded C00-C97) [14], matching ‘malignancies’ in new diagnosed cancer registrations [3], in each adult age band given in rates per million [pm] persons. This enables comparisons to be made between countries of differing size and to produce a percentage of change, a method successfully used in other comparative international studies [e.g. 6, 19, 20]. The baseline years are three-year average for 1979–81, compared with the index three-year average for 2000–2, and percentage changes of 0.10 (10%) have been defined as substantial, but as in previous international studies, substantial is defined here as plus or minus 0.20 (20%).

Cancer mortality by sex is reported for each decade age band and an average rate for the 15–74-year age group was calculated, where it might be thought that cancer services would make the most significant impact on death rates [9, 21]. However, as cancer mortality in younger people is relatively low, the ‘younger’ age bands 15–24 and 25–34 are combined into a young adult band 15–34 and then each separate decade age band from 35–44 to 75+.

Spearman rank-order correlation was used to determine how consistent the changing average rates were over time, by sex, between the ten MDCs.

The three-year average baseline years 1979–81 were chosen as all MDCs were using the same International Classification of Disease (ICD) editions as for the latest three-year average index years 2000–2 [18], which allows for comparisons of global mortality categories over different periods [22]. The earliest baseline for ‘Germany’ was for 1980–2, also, some countries’ index data ended earlier, i.e. Canada and France 1998–2000. Whilst WHO data for the United States also ended in 2000, national figures were available and the three-year average data were calculated for the period of 2000–2 [23].

Data for England and Wales are for 2000–2, but earlier (1998–2000 and 1999–2001) Anglo-Welsh figures will be shown to enable coincident temporal comparison to match those other countries with these end points. Mortality rates by age bands and sex will be compared between the baseline and index years to provide the percentage change. Chi-squared tests compared the outcomes between England and Wales and the other MDCs and take as ‘statistically significant’ probability levels <0.05, an approach that has been used elsewhere [6, 19, 20]. As the actual rates are relatively small, to avoid any errors due to statistical artefacts and multi-testing between age bands, any results falling just short of statistical significance will not be reported.

It should be noted that because the behaviour of women over the past 20 or more years has converged with that of men in terms of behaviour, e.g. smoking and employment, there is a need to consider each age band for each sex [24, 25].

*Eligible countries:*contrasting small with large populations can be problematic, therefore only countries with populations in excess of 15 million were reviewed and designated as a ‘major developed country’. However, because of their special circumstances and/or the absence of consistent data the larger Warsaw pact countries, Africa and Latin American have not been included in this study.

The eligible MDCs include: Australia, Canada, England and Wales, France, Germany, Italy, Japan, the Netherlands, Spain and the United States, which have some of the highest GDPEH rates in the world [16].

In the tables, the data referring to England and Wales is rank ordered in relation to the other MDCs over the two periods in terms of rates of each age band, 1 being the highest and 10 being the lowest.

## Results

### The problem—changing incidence in England and Wales

Table 1 shows the percentage increase between the averages for 1979–80 and 2003–4 for all registrations and all malignancies. Notable increases in all registrations for female youth (15–24) and young adults (25–34) were up by 425% and 164%, respectively.

The male All Age rate rose by 48% and female by 51% over the period. Except for the age bands 0–4 and 25–44, the female rates rose more than the male rates, and some notable increases amongst females were: youth up to 66% and the over-55s rose by more than 40% averaging an annual increase of 1.9%. A positive significant correlation was found for changing all registrations by age and sex (p < 0.025), but the positive correlation for the malignancies was not statistically significant (p < 0.1), indicating less convergent malignancy rates between the sexes.

### Responding to the problem: MDC GDPEH

Table 2 gives each of the MDC fiscal response to health problems seen in the GDPEH between 1980 and 2003 and an average for the period.

Throughout, the United States had the highest GDPEH, rising from 9.1% to 15%, whilst the United Kingdom went from 5.6% to 9.3%, moving from ninth to eighth position of the ten MDCs, but continued to be below the MDC average of 9.85%, despite record rises in the last five years.

However, it was found that over the period, the United Kingdom had the highest increase in GDPEH (66%), compared to an average rise of 39% in the other MDCs.

Over the period, there was a very significant correlation of the increasing rates of GDPEH amongst the ten MDCs (p < 0.001), indicating consistency over time.

### Outcomes—cancer death rates: males (Table 3)

Initially, the average male rate (15–74) in England and Wales, 4029 per million, was the third highest, but by the end of the period, this rate of 3062 per million was now eighth, only Australia and Japan being lower.

The table also shows the rank order of the rates of average deaths for the two periods for every MDC and rank order for the Anglo-Welsh rates in each age band. The changing average rates were positively but not significantly correlated.

*Age bands:* In every MDC, except France and Spain, the 15–44-year-old male age band death rates fell substantially (>20%), and for the 45–54 group every MDC declined substantially except Spain and Japan. England and Wales’ rates had some of the biggest falls.

There were substantial declines amongst the 54–64-year-olds in England and Wales (29%), the Netherlands (25%), and Australia and Italy (22%), whilst England and Wales’ and the Netherlands’ 65–74-year-old male rates fell by 22% and 21%, respectively.

There were no substantial falls in the 75+ group, with notable rises in Italy 22%, Japan 38% and Spain 25%.

*Females* (Table 4): The highest average female rates in both periods were found in England and Wales, initially 2716 per million and, by 2000–2, 2359 per million, although these were the only rates to fall substantially (>20%) amongst all the MDCs.

Female changing average rates were significantly correlated, showing consistency across the MDCs between the periods (p < 0.001).

*Age bands:* every country’s 15–34 female rates fell substantially except the Netherlands (down only 16%), with substantial falls in the 35–44 groups. The biggest drop was in England and Wales, by 34%.

In the female 45–54 age band, only Australia, Canada and England and Wales had substantial falls and in only England and Wales did the 5–-64-year-old female age band see a substantial decrease, down 22%.

With respect to the 75+ age band, there were no substantial reductions in any MDC but a notable increase in France (51%).

### Declining cancer deaths and increased GDPEH

Table 5 shows the average death rates proportionately for each country’s GDPEH for 1979–81 and 2000–2 by sex and the combined (men and women) percentage of reduced cancer mortality, juxtaposed against the percentage of increases in each country’s GDPEH.

It can be seen that the two MDCs with the biggest proportional increases in GDPEH, England and Wales and the United States, 66% and 65%, respectively, also had the biggest reduction in cancer deaths, 52% and 47%. Conversely, the two MDCs with the smallest reduction in mortality, Italy and Japan at 27% and 28%, respectively, also had the smallest increases in their GDPEH, 20% and 22%, between the two periods. There was a very significant correlation (p < 0.001) showing an association between relative increased spending on health and a reduction in cancer deaths.

### International comparisons (1979–2002)

Table 6 shows the significant p values based upon the chi-squared test, comparing England and Wales with every other MDC, which improved significantly more than the other MDCs unless marked by #, indicating that the Anglo-Welsh rates did not fall as much.

*Males:* between the two periods, the (15–74) male average rates declined statistically significantly more in England and Wales than in any other MDC, except the Netherlands.

With regard to the 35–44-year-olds, the Anglo-Welsh male death rates declined significantly more than that of France and Spain, and the 45–54 age group did better than in every MDC except Australia, Canada and Italy.

For 55–64 years old, the Anglo-Welsh rates declined significantly more than every MDC, and for 65–74-year-olds, more than all countries, except the Netherlands.

For the 75+ rates, the picture was more mixed, but England and Wales had bigger reductions than had France, Italy, Japan and Spain.

*Females:* Female average rates in England and Wales declined more than in Canada, France, the Netherlands and the United States.

For the 35–44 age group, rates in England and Wales reduced significantly more than every MDC except Italy and Japan, and for the 45–54 age group, rates in England and Wales were significantly better than every MDC except Australia and had significantly bigger reductions for the 55–64 age group compared to the other MDCs, with the exception of Japan.

With regard to the 65–74 age band, the picture was mixed, England and Wales had better outcomes than Canada, France and the United States, but rates in Germany, Italy and Japan fell significantly more than those in England and Wales.

For the 75+ age group, England and Wales did significantly better than did France, whilst the Netherlands had a better outcome.

### Gender variations in the major developed countries: (1979–2002)

It was noted that in the majority of the MDCs, male rates of cancer deaths fell more than women’s, with the exception of Japan and Spain. Table 7 shows the significant p values when comparing male rates with female deaths for the corresponding MDC. In all cases, except for countries marked with #, significantly greater reductions were observed for men than for women, with # indicating a better outcome for women.

Average male rates declined significantly more than the average female in Australia, England and Wales, the Netherlands and the United States (15–74 age group). Conversely, in Japan and Spain, female rates fell significantly more than their male counterparts, also in the age bands 55–75+.

Amongst the 45–54 age group, males did better than the females in Italy, Japan, the Netherlands and the United States; in the 55–64 age group, in Italy and the Netherlands; but the reverse was true in Japan for the 54–75+, where women had the biggest reductions.

Amongst the 65–74 years old, male rates declined more in Australia, England and Wales, the Netherlands and the United States, and, for the 75+ in England and Wales, France and the United States.

## Conclusions

One limit to the study is the slight difference in index years, and the fact that the United States figures required supplementation [23], but the main weakness was that we could not find reasonably up-to-date new incidence figures for the other MDCs to match those of England and Wales [3]. Notwithstanding this limitation, the study provides a broad reliable indicator of the differences in cancer mortality between the two periods of 1979–81 and 2000–2 in the MDCs considered, within the context of national spending on health care [16].

The hypothesis that there would be no significant differences between England and Wales and the other nine MDCs for malignancy deaths between the periods can generally be rejected for men and to a lesser extent also for women, as the Anglo-Welsh male average (15–74) rates declined significantly more than every MDC except for the Netherlands, whilst Anglo-Welsh women did significantly better than Canada, Japan, the Netherlands and the United States.

The hypothesis that there would be no significant difference between gender rates can also be rejected for Australia, Canada, England and Wales, Italy, the Netherlands and the United States, where male rates declined more than female rates, whilst the reverse was true for Japan and Spain.

Finally, the hypothesis that there would be no significant association between reduced cancer deaths and proportional increases in GDPEH can be strongly rejected.

However, we cannot explain the changes found and country-specific research is required. There are a number of further intriguing findings:

First, in general, male cancer deaths are higher than female, except in women aged 35–44, in eight MDCs. And for the 45–54-year-old age group in seven MDCs, France and Spain being the exceptions.

The second gender related finding is, with the exception of Japan and Spain, cancer deaths for men declined significantly more than for women, suggesting the impact of life-style changes on women, with more women entering the work force [24,25], which should have major implications for future policies and planning of services.

Third, whilst all countries had substantial reductions in cancer deaths, indicating advances in care and treatment, the Anglo-Welsh did particularly well.

Fourth, it has been found that cancer survival rates are influenced by increased expenditure, including the use of newer, and invariably more costly, anti-cancer drugs [9, 21,26–28] set within the context of each MDC substantially raising its GDPEH.

Despite the recent increase in the GDPEH of England and Wales (9.3%), it remains below the MDC average (9.85%), and only Japan and Spain spent less over the same period. Nonetheless, the Anglo-Welsh GDPEH increase was the highest amongst the MDCs, and the correlation between a reduction in cancer deaths and increased national expenditures on health, should encourage governments to respond to the challenge.

Finally, the reduction in malignancy deaths in all the MDCs, especially amongst the under-65s, should be a boost for patient morale, their families and frontline staff in the MDCs and in England and Wales in particular. However, this encouraging improvement should not distract from the increased incidence of cancer [1–3], especially in England and Wales, as well as the continuing negative link with socio-economic factors [29,30].

So, whilst it may be true to say that the treatment of cancer has never been better, still more needs to be done, especially when facing the challenges posed by the increasing incidence of malignancies in the general population.

## Figures and Tables

**Table 1:**
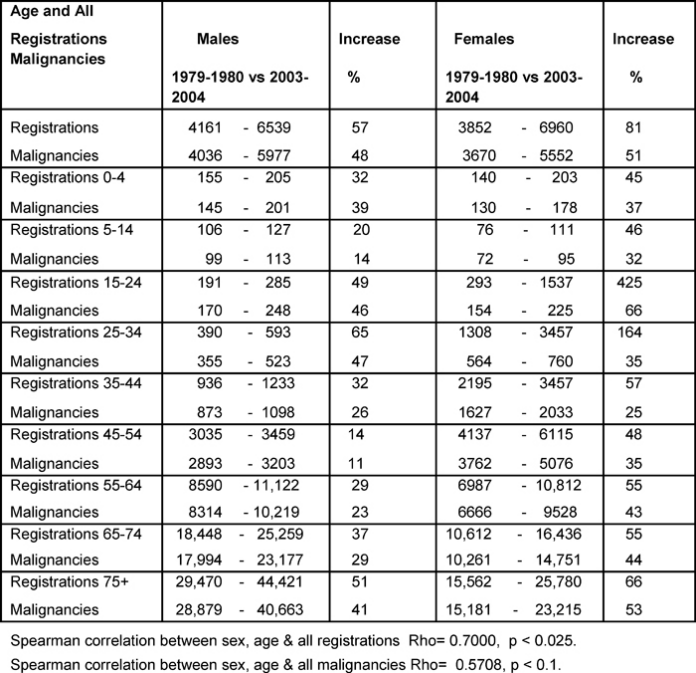
Percentage increases in registrations of newly diagnosed cancers and malignancies by age and sex 1979–80 versus 2003–4 (rates per million)

**Table 2:**
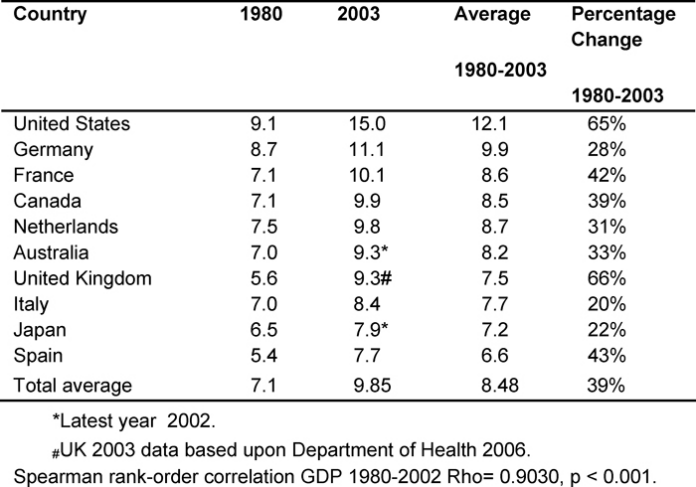
Total percentage of GDP expenditure on health by country 1980–2003 (countries ranked by highest current GDPEH)

**Table 3:**
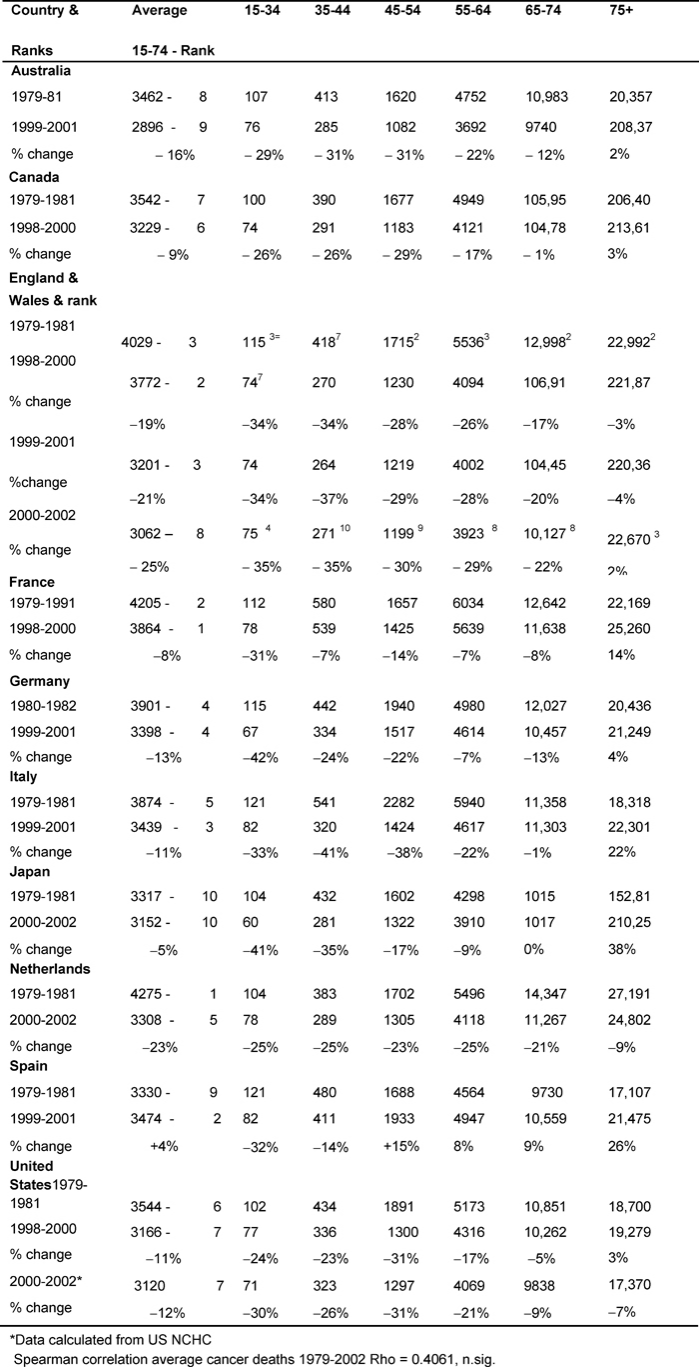
Male all malignancy deaths by age in MDC rates per million and percentage of change 1979–2002 (England and Wales rank ordered compared to MDCs, 1 being highest rate)

**Table 4:**
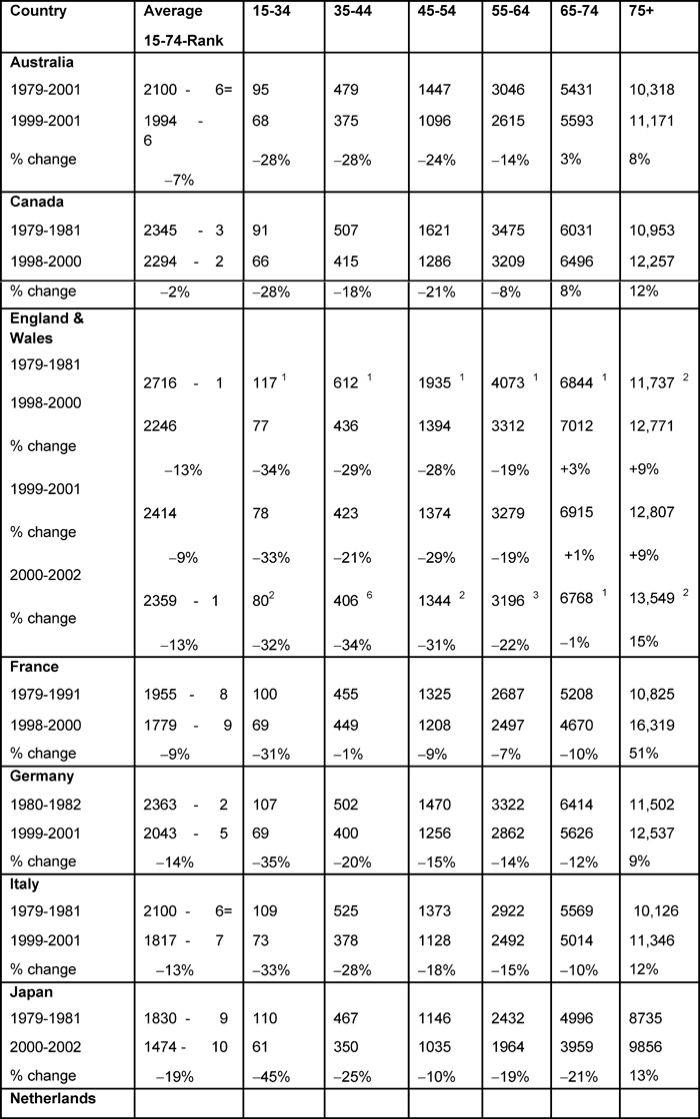

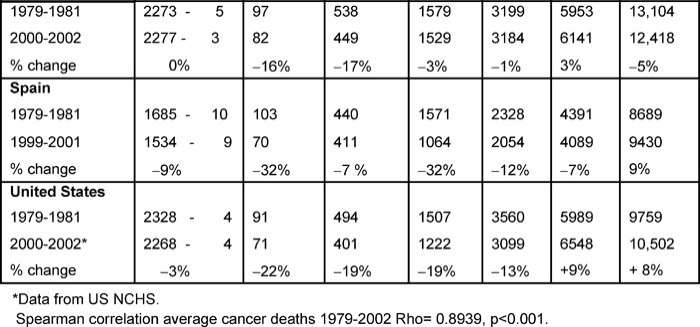
Female all malignancy deaths by age in MDC rates per million and percentage change in 1979–2002 (England and Wales rank ordered compared to MDC, 1 being highest rate)

**Table 5:**
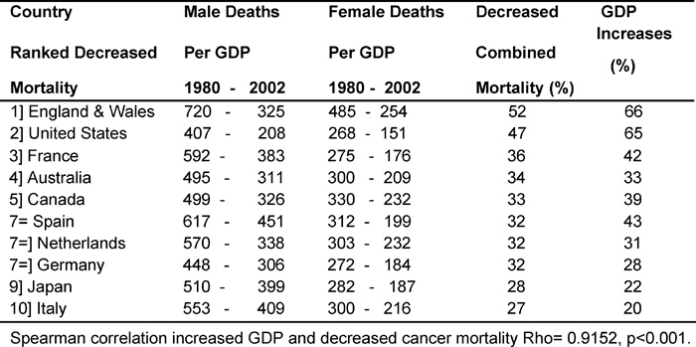
Decreased cancer deaths (men and women) and increases in GDPEH 1980–2002. (Countries ranked by decreased mortality—rates per million)

**Table 6:**
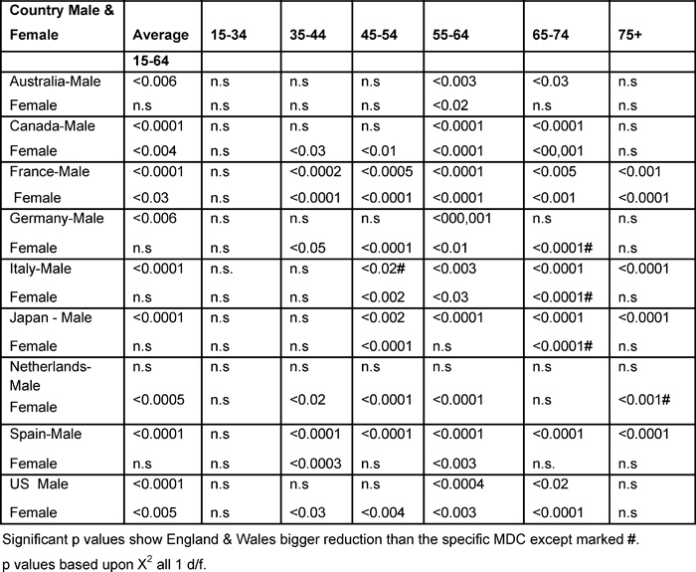
Level of significant change of England and Wales rates compared with each MDC 1979–2002 by age and sex (p level of significant change)

**Table 7:**
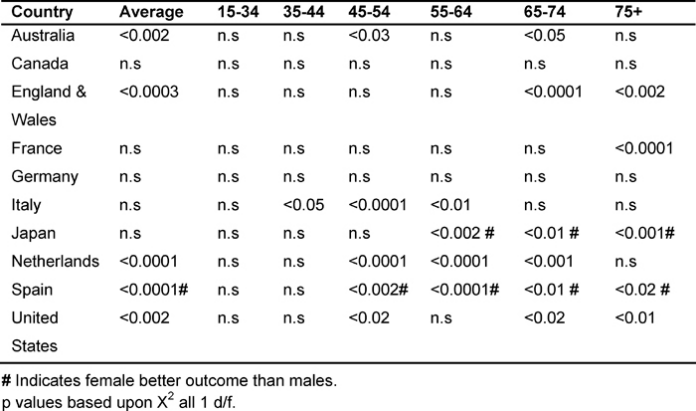
Comparing male versus female rates in each MDC by age 1979–2002 (males better outcome unless # indicating females better outcome)
